# 1202. A Multi-Center Validation of the Electronic Health Record ‘Admission Source’ Field Against Clinical Notes for Identifying Hospitalized Patients with Long-term Care Facility Exposure

**DOI:** 10.1093/ofid/ofac492.1035

**Published:** 2022-12-15

**Authors:** Katherine E Goodman, Monica Taneja, Laurence Magder, Philip Resnik, Mark Sutherland, Scott Sorongon, Eili Klein, Pranita Tamma, Anthony Harris

**Affiliations:** University of Maryland School of Medicine, Baltimore, Maryland; University of Maryland School of Medicine, Baltimore, Maryland; University of Maryland School of Medicine, Baltimore, Maryland; University of Maryland College Park, College Park, Maryland; University of Maryland School of Medicine, Baltimore, Maryland; University of Maryland School of Medicine, Baltimore, Maryland; Johns Hopkins School of Medicine, Baltimore, Maryland; Johns Hopkins School of Medicine, Baltimore, Maryland; University of Maryland School of Medicine, Baltimore, Maryland

## Abstract

**Background:**

A current or recent long-term care facility (LTCF) stay is a strong risk factor for antibiotic-resistant bacterial colonization and infection. However, most electronic health record (EHR) systems do not systematically record LTCF exposure. Absent manual chart review, which is resource-intensive and cannot be incorporated into automated screening algorithms, there is no definitive method for identifying LTCF-exposed inpatients. As a surrogate, researchers often use ‘Admission Source’ to identify LTCF transfers, but this EHR field has not been previously validated, and it may miss the unknown percentage of patients with recent, but not current, LTCF stays. This study evaluated the accuracy of ‘Admission Source’ in identifying LTCF-exposed inpatients.

**Methods:**

This was a retrospective study of adult admissions from 2018 – 2021 across 12 hospitals in the University of Maryland Medical System. We extracted patient and encounter data and classified patients as LTCF-exposed by ‘Admission Source’ if they were LTCF transfers. For 315 randomly sampled admissions, M.T. and K.G. reviewed the admission ‘History & Physical’ note for mention of LTCF exposure (Fig. 1). Assuming an indication of LTCF in either the ‘Admission Source’ field or clinical note represented true exposure, we estimated each method’s sensitivity with 95% confidence intervals.

**Figure:**
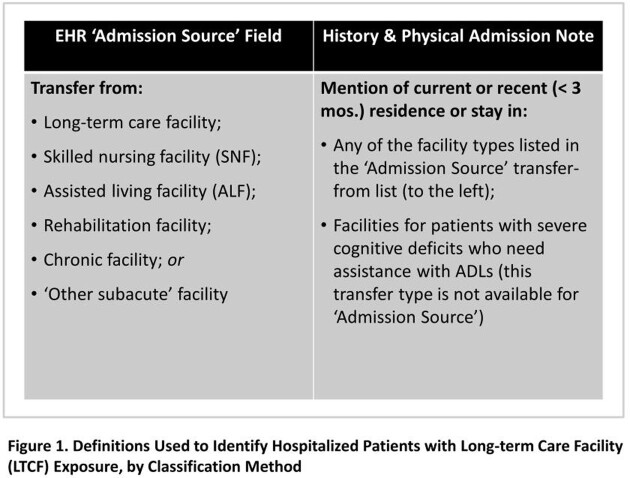

**Results:**

Across 280,581 admissions, 9,476 (3.4%) had an ‘LTCF transfer’ admission source. In the validation sample, 26 (8.3%) were classified as LTCF-exposed by either ‘Admission Source’ or clinical note, of which 12 were identified in the ‘Admission Source’ field and 25 in the notes (Fig. 2). The sensitivity of ‘Admission Source’ for detecting LTCF exposure was 46% (29% – 65%) and for clinical notes was 96% (81% - 99%). Most (12/14) patients missed by ‘Admission Source’ were current LTCF residents (Fig. 3).

**Figure:**
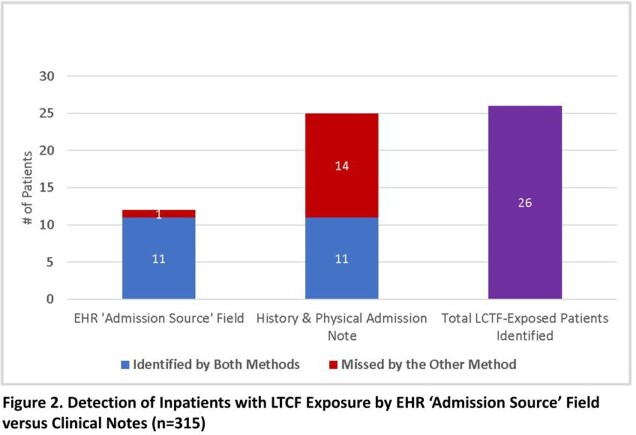

**Figure:**
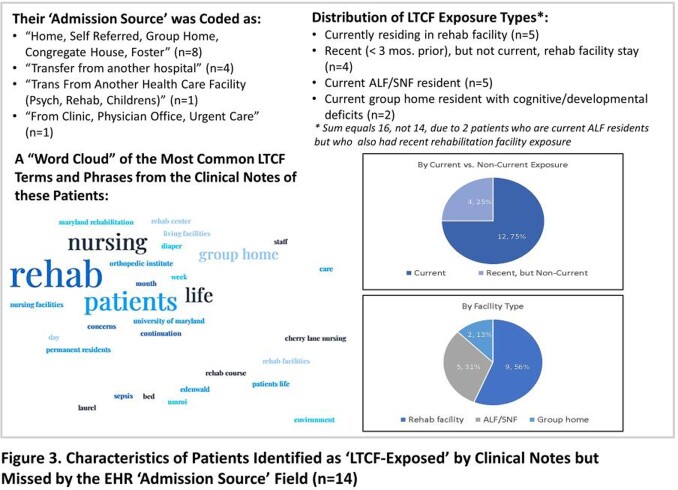

**Conclusion:**

The EHR ‘Admission Source’ field misses the majority of inpatients with recent or current LTCF exposure, risking substantial misclassification in research studies and clinical algorithms that incorporate this variable. Automated techniques for analyzing free-text notes, such as natural language processing, could significantly improve detection of these patients to assist hospital epidemiology and infection control efforts.

**Disclosures:**

**All Authors**: No reported disclosures.

